# Glioblastoma survival prediction through MRI and clinical data integration with transfer learning

**DOI:** 10.1007/s11548-025-03548-1

**Published:** 2025-12-04

**Authors:** A. Marasi, D. Milesi, D. Aquino, F. M. Doniselli, R. Pascuzzo, M. Grisoli, A. Redaelli, E. De Momi

**Affiliations:** 1 Department of Electronic, Information and Bioengineering, Politecnico di Milano, Milan, Italy; 2 Neuroradiology Unit, Fondazione IRCCS Istituto Neurologico Carlo Besta, Milan, Italy

**Keywords:** Glioblastoma, Overall survival prediction, Deep learning, Multimodal MRI

## Abstract

**Purpose:**

Accurate prediction of overall survival (OS) in glioblastoma patients is critical for advancing personalized treatments and improving clinical trial design. Conventional radiomics approaches rely on manually engineered features, which limit their ability to capture complex, high-dimensional imaging patterns. This study employs a deep learning architecture to process MRI data for automated glioma segmentation and feature extraction, leveraging high-level representations from the encoder’s latent space.

**Methods:**

Multimodal MRI data from the BraTS2020 dataset and a proprietary dataset from Fondazione IRCCS Istituto Neurologico Carlo Besta (Milan, Italy) were processed independently using a U-Net-like model pre-trained on BraTS2018 and fine-tuned on BraTS2020. Features extracted from the encoder’s latent space represented hierarchical imaging patterns. These features were combined with clinical variable (patient’s age) and reduced via principal component analysis (PCA) to enhance computational efficiency. Machine learning classifiers—including random forest, XGBoost, and a fully connected neural network—were trained on the reduced feature vectors for OS classification.

**Results:**

In the four-modality BraTS4CH setting, the multi-layer perceptron achieved the best performance (F1 = 0.71, AUC = 0.74, accuracy = 0.71). When limited to two modalities on BraTS2020 (BraTS2CH), MLP again led (F1 = 0.67, AUC = 0.70, accuracy = 0.67). On the IRCCS Besta two-modality cohort (Besta2CH), XGBoost produced the highest F1-score and accuracy (F1 = 0.65, accuracy = 0.66), while MLP obtained the top AUC (0.70). These results are competitive with—and in some metrics exceed—state-of-the-art reports, demonstrating the robustness and scalability of our automated framework relative to traditional radiomics and AI-driven approaches.

**Conclusion:**

Integrating encoder-derived features from multimodal MRI data with clinical variables offers a scalable and effective approach for OS prediction in glioblastoma patients. This study demonstrates the potential of deep learning to address traditional radiomics limitations, paving the way for more precise and personalized prognostic tools.

## Introduction

Glioblastoma multiforme (GBM) is the most prevalent and aggressive primary brain tumor, characterized by rapid progression and poor prognosis. Standard treatment involves maximal safe surgical resection, radiotherapy, and temozolomide chemotherapy; however, most patients experience recurrence, with a median overall survival (OS) of approximately 15 months [1, 2].

Accurate OS prediction is crucial for optimizing patient management, enabling early identification of high-risk cases and personalized treatment planning [3]. Unfortunately, prognostic modeling is hindered by inconsistent survival endpoints, variable imaging protocols, and heterogeneous computational workflows, limiting reproducibility and clinical application.

Molecular biomarkers, such as IDH1/2 mutations and MGMT promoter methylation, are valuable for OS prediction [4, 5]. MGMT methylation enhances temozolomide efficacy, improving survival outcomes [5], while IDH1 mutations are associated with a median OS of 31 months compared to 15 months for wild-type glioblastomas [6]. Although these biomarkers provide stratification accuracies of up to 75%, their invasive nature limits widespread adoption [7, 8].

Non-invasive imaging techniques, especially magnetic resonance imaging (MRI), offer an alternative for OS prediction by evaluating imaging-derived features, including tumor volume, necrosis, and edema [9–11]. Such features serve as non-invasive biomarkers and support advanced computational approaches like radiomics and deep learning, which extract high-dimensional patterns to enhance prognostic accuracy [8, 12].

Radiomics has demonstrated promise in predicting OS in glioblastoma patients. Bae et al.[13] demonstrated that integrating radiomic features with genetic and clinical profiles significantly improved the accuracy of OS predictions (integrated AUC = 0.76). Similarly, Li et al. [14] used preoperative MRI-based radiomics to achieve an AUC of 0.76, linking radiomic features to macrophage infiltration in the tumor microenvironment.

Despite these promising advancements, radiomics faces several limitations [15, 16]. A primary limitation is that radiomics features must be manually selected and engineered, requiring expert domain knowledge to identify relevant attributes such as texture, shape, or intensity. This manual process is inherently subjective and risks overlooking complex or non-intuitive patterns in imaging data that may be critical for accurate predictions. Additionally, variability in imaging protocols, preprocessing workflows, and feature selection methods reduces reproducibility and limits the generalizability of radiomics models.

Emerging Artificial Intelligence (AI) techniques, particularly machine learning (ML) and deep learning (DL), offer transformative solutions by automating feature extraction, reducing dependence on manual feature selection, and uncovering complex imaging patterns [17, 18]. Pei et al. [19] employed a hybrid approach that combined deep learning and traditional regression models. Features were extracted using convolutional neural networks (CNN) and integrated with patient age in a linear regression model for survival prediction. This method achieved an accuracy of 48.4% on the test set and 58.6% on the validation set, illustrating the potential and challenges of combining imaging features with clinical data. Chato et al. [20] integrated tumor segmentation with machine learning classifiers, achieving a 68.8% accuracy in stratifying patients into two-class survival categories.

Other approaches merge deep learning with classical learning techniques. Sun et al. [21] developed a hybrid framework combining deep learning-based tumor segmentation and radiomic feature extraction from multimodal MRI scans, integrated with clinical features, to predict survival in glioblastoma patients. Their approach achieved a 61% accuracy in classifying patients into short-, mid-, and long-term survival groups during the BraTS 2018 challenge. McKinley et al. [22] combined a deep learning-based segmentation model with a random forest classifier, incorporating features such as tumor core segmentation and age, achieving an accuracy of 61.7% in classifying patients into short-, mid-, and long-term survival groups.

Most recently, Valbuena Rubio et al. [23] employed transfer learning using pre-trained models, such as DenseNet121, for survival prediction. Their approach achieved a balanced accuracy of 65% for classifying patients into short-, mid-, and long-term survival categories, demonstrating the effectiveness of transfer learning in addressing the challenges posed by limited datasets.

These AI-driven methodologies demonstrate the transformative potential of automated, multimodal approaches for OS prediction.

This study proposes a framework that leverages a pre-trained U-Net-like architecture to extract high-dimensional features directly from the encoder’s latent space, overcoming the reliance on manually designed radiomic features. This method employs transfer learning (pre-trained on BraTS2018 dataset) to capture complex imaging patterns from MRI scans in the BraTS2020 and Fondazione IRCCS Istituto Neurologico Carlo Besta datasets. By integrating these latent features with a clinical variable, the framework provides a robust pipeline for glioblastoma overall survival prediction, demonstrating adaptability across diverse datasets and addressing key limitations of traditional radiomics in feature engineering and generalizability.

## Methods

**Fig. 1:**
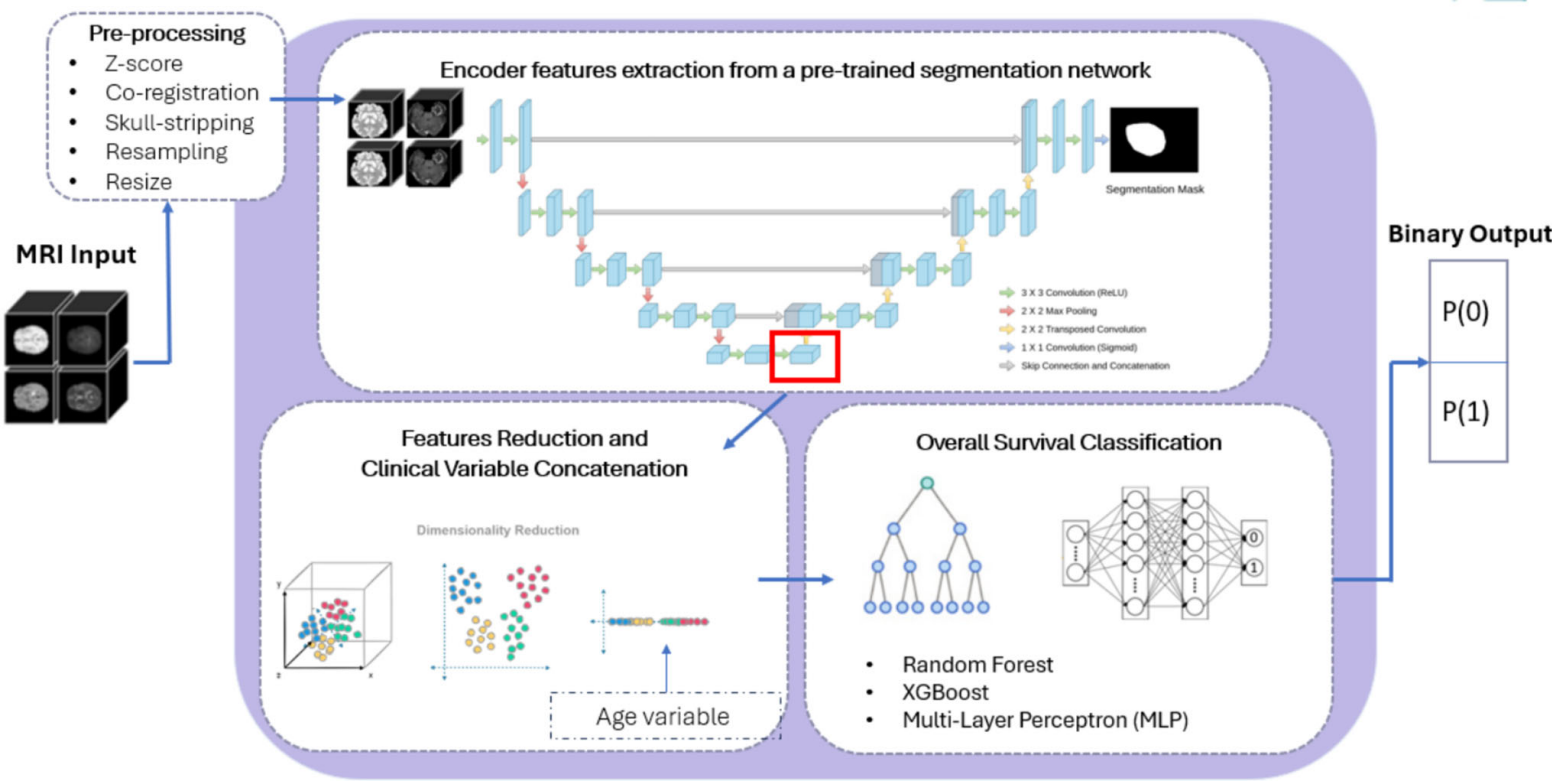
Proposed pipeline for glioma feature extraction and survival prediction. Multimodal 3D MRI volumes are pre-processed according to dataset requirements, processed by a 3D SegResNet for feature extraction, and reduced via PCA. The resulting features, combined with clinical data, are used for survival classification

The proposed pipeline (Fig. 1) for GBM overall survival prediction integrates imaging and clinical data through a multi-step process. Multimodal MRI volumes are preprocessed and transferred to a 3D SegResNet for tumor segmentation [24]. High-dimensional features are extracted from the encoder’s latent space, capturing relevant spatial patterns from the MRI data. These features are then reduced via principal component analysis (PCA) to retain the most informative components.

The reduced imaging features, combined with age as clinical variable, are input into three machine learning classification models: (i) random forest, (ii) XGBoost, and (iii) a multi-layer perceptron (MLP). This framework leverages advanced imaging analysis and machine learning to provide precise prognostic predictions for glioblastoma patients.

### Input MRI 3D volume

Two distinct datasets are used to evaluate the performance of the proposed framework for OS prediction: the BraTS2020 dataset and a proprietary dataset from the Fondazione IRCCS Istituto Neurologico Carlo Besta (Milan, Italy). Both datasets provided imaging and clinical data essential for glioma segmentation and survival analysis. A detailed summary of the datasets will be provided in the subsequent sections. Table 1 outlines the technical specifications of the MRI data, including imaging modalities, resolution, and available segmentation masks. Table 2 provides an overview of the clinical data, summarizing key attributes such as patient demographics and overall survival outcomes, offering a comprehensive basis for the analysis.

**Table 1:** Technical specifications of MRI data used in this study

Dataset	MRI Sequences	Spacing [mm$^3$]	Size [pixels]	Annotations
BraTS2020	T1, T1Gd, T2, FLAIR	$1\times 1\times 1$	$224\times 224\times 144$	ET, ED, NEC
IRCCS Besta	T1Gd, FLAIR	$1\times 1\times 1$	$224\times 224\times 144$	ET, ED, NEC

*T1* T1-weighted imaging, *T1Gd* T1-weighted post-gadolinium (contrast-enhanced) imaging, *T2* T2-weighted imaging, *FLAIR* fluid-attenuated inversion recovery, *ET* enhancing tumor, *ED* edema, *NEC* necrotic tumor. Voxel spacing and dimensions for IRCCS Besta Dataset are expressed as mean values due to the variability of the data

**Fig. 2:**
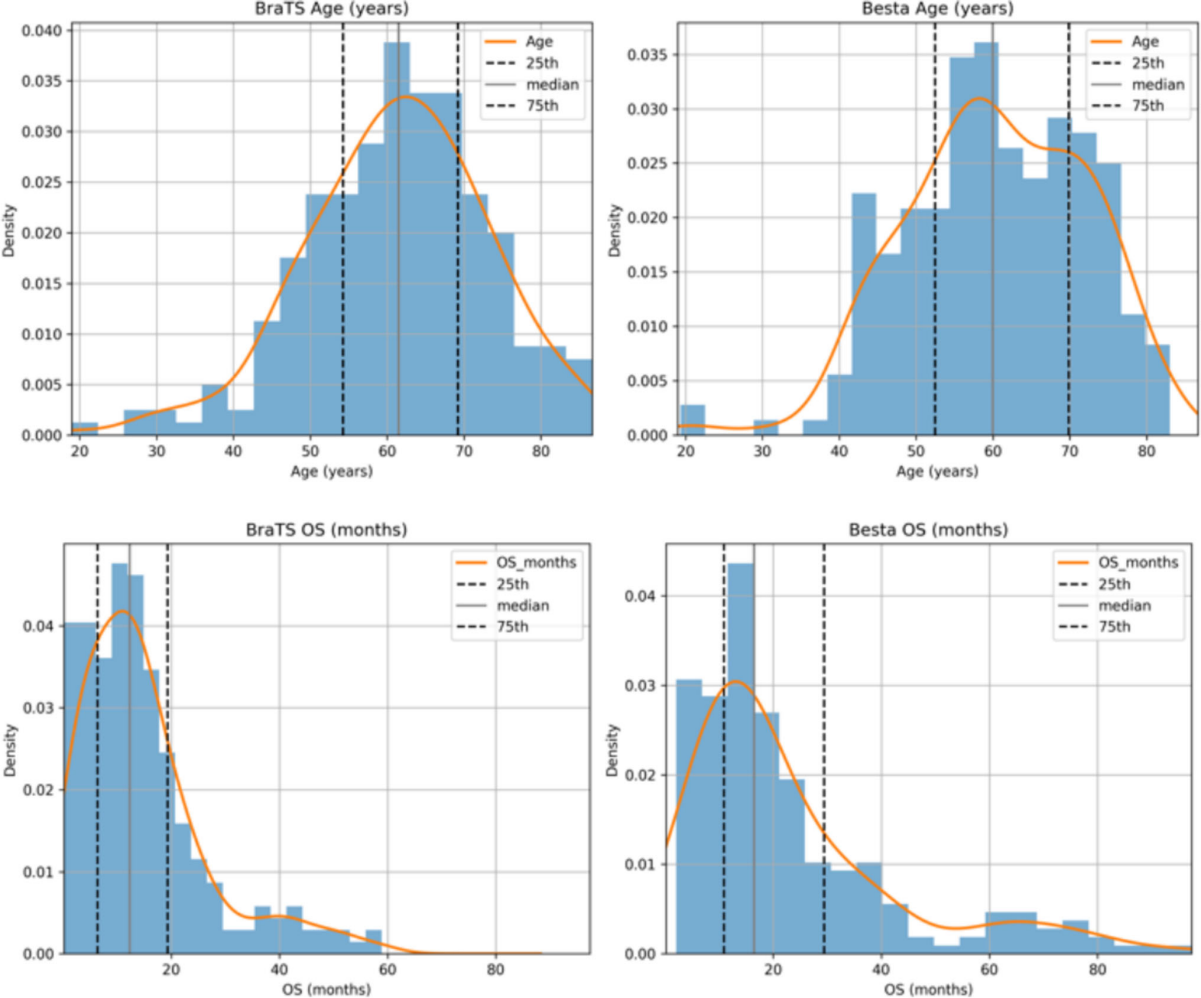
Distribution of age at diagnosis and overall survival (OS) for the two cohorts. Upper row: age histograms with median and percentiles for (left) BraTS data and (right) IRCCS Besta data. Lower row: OS (in months) distributions with median and percentiles for (left) BraTS data and (right) IRCCS Besta data

Clinical data are presented as median ± standard deviation (Table 2) and their distributions are illustrated in Fig. 2.

**Table 2:** Technical specifications of clinical data used in this study

Dataset	Patient cohort	Age (years) [IQR]	Overall survival (months) [IQR]
BraTS2020	236	61.5 [54.2–69.2]	12.3 [6.3–19.3]
IRCCS Besta	228	60.0 [52.5–69.9]	16.5 [10.9–29.5]

Data are presented as median [$25_{\textrm{th}} - 75_{\textrm{th}}$ percentile]

#### BraTS2020 dataset

**Fig. 3:**
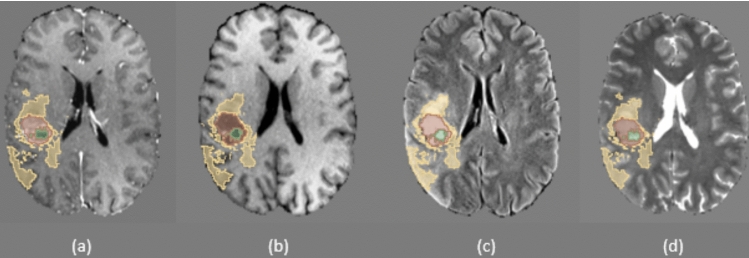
Example slices from the BraTS 2020 MRI dataset. **a** T1-weighted post-gadolinium (contrast-enhanced) (T1Gd); **b** T1-weighted imaging (T1); **c** T2-weighted imaging (T2); **d** T2 fluid-attenuated inversion recovery (T2-FLAIR). The segmentation mask is shown on the far right, with the following labels: green (NEC, necrotic tumor core), yellow (ED, peritumoral edema), and red (CE, contrast-enhancing tumor)

The BraTS 2020 dataset is part of the Multimodal Brain Tumor Segmentation Challenge. It comprises pre-operative MRI scans of 369 patients acquired from multiple institutions using a variety of MRI scanners and protocols. Each case includes four MRI sequences—T1, T1Gd, T2, and T2-FLAIR—and tumor annotations identify enhancing tumor (ET), peritumoral edema (ED), necrotic and non-enhancing tumor core (NEC) regions, with expert-validated ground truth segmentations (Fig. 3). All images have been co-registered on the same anatomical template, interpolated to uniform isotropic resolution of 1 mm$^3$ and dimension of $240\times 240\times 144$ as reported in Table 1, and finally skull-stripped.

In addition to imaging data, the dataset provides clinical metadata, including overall survival (OS) and patient age. For survival prediction, a subset of 236 cases with complete OS labels and corresponding imaging data was selected. This ensured compatibility with the study’s aim of robust training and evaluation of predictive models.

#### IRCCS Besta dataset

**Fig. 4:**
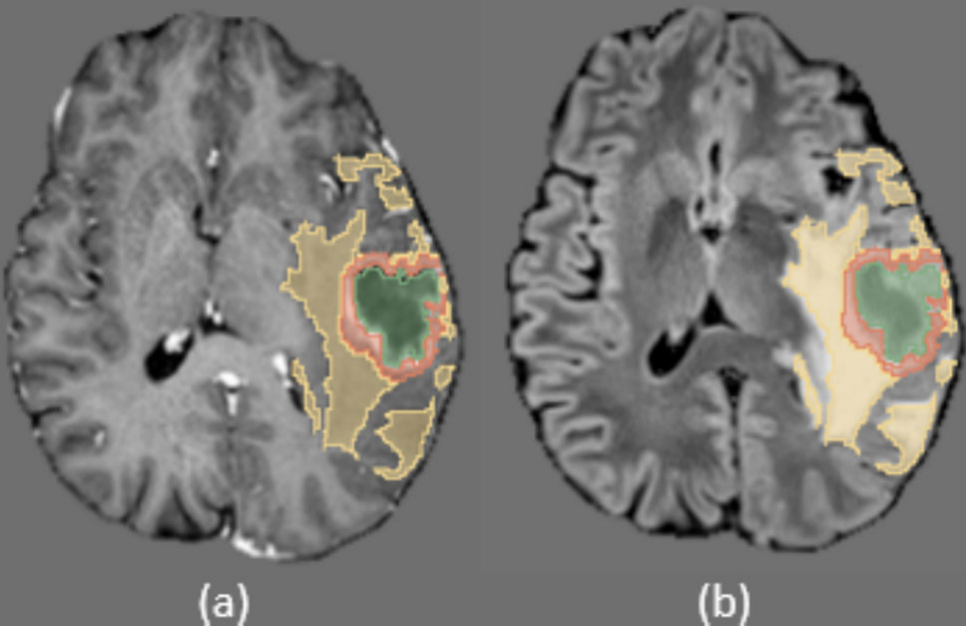
Example slices from the IRCCS Besta MRI dataset. **a** T1-weighted post-gadolinium (contrast-enhanced) MRI (T1Gd); **b** 2 fluid-attenuated inversion recovery (T2-FLAIR). The segmentation mask is shown on the far right, with the following labels: green (NEC, necrosis), yellow (ED, peritumoral edema), and red (CE, contrast-enhancing tumor)

The IRCCS Besta dataset includes MRI scans from 420 glioma patients, collected retrospectively at the IRCCS Besta Neurological Institute of Milan. Imaging data were acquired using either Siemens MAGNETOM Avanto or Philips Ingenia 1.5 T MRI scanners. For each patient, volumetric T1Gd and FLAIR sequences were provided, with a section thickness of 1 mm (Fig. 3). Ground-truth tumor annotations include necrosis (NEC), contrast-enhanced regions (CE), and hyperintense regions (ED), labeled by expert neuroradiologists (Fig. 4; Table 3).

Clinical metadata such as OS and patient demographics are also available, ensuring compatibility for survival prediction tasks (Table 2). To ensure alignment with available OS data and focusing exclusively on high-grade gliomas, a subset of 228 patients was ultimately selected for downstream analyses.

#### Class distribution for OS prediction

For survival prediction, overall survival was encoded as a binary variable based on a threshold of 400 days (13 months). Cases with survival times $\le$ 400 days were labeled as “short survival” (Class 0), while those > 400 days were labeled as "long survival" (Class 1). This threshold represents a trade-off between clinical relevance and class balance: it aligns with established survival benchmarks while yielding well-separated groups of comparable size.

To train and evaluate the predictive framework, the dataset was split into training and testing sets using a 70–30 ratio. The training set was used to develop the machine learning models, while the independent test set provided a reliable assessment of performance. Table 3 summarizes the class distribution for each dataset and provides details of the training and testing splits.

**Table 3:** Class distribution and dataset split for OS prediction among the two datasets

Dataset	Class 0	Class 1	Training set	Test set
BraTS2020	117	119	165	71
IRCCS Besta	79	149	159	69

#### Pre-processing and data preparation

Pre-processing was essential to minimize variability introduced by differing imaging protocols, scanner characteristics, and acquisition settings across datasets. Both datasets underwent pre-processing to ensure consistency and compatibility with the 3D SegResNet model, including a resizing of all images to a standardized spatial dimension of $224\times 224\times 144$.

The BraTS2020 dataset provided pre-processed images, except for intensity normalization, ensuring standardized inputs across scans. In contrast, the IRCCS Besta dataset required additional pre-processing to align with the BraTS2020 specifications. Steps included multi-modal imaging co-registration, skull-stripping, resampling and intensity normalization. All these steps were performed via known softwares for brain MRI manipulation; in particular, multi-modal image registration was performed using the Elastix [25] package, skull-stripping was carried out with the HD-BET [26] tool, and image resampling was executed via the SimpleITK [27] library’s ResampleImageFilter.

**Fig. 5:**
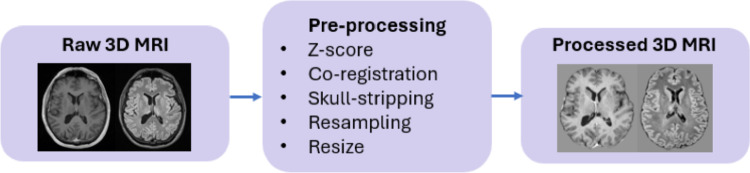
Preprocessing workflow applied on 3D MRI volumes of IRCCS Besta dataset

A flowchart of the preprocessing workflow applied to the IRCCS Besta dataset is shown in Fig. 5.

The 3D SegResNet model is designed to process input volumes of fixed dimensions (224 $\times$ 224 $\times$ 144). To ensure compatibility, all MRI volume from both datasets were uniformly cropped to these dimensions during pre-processing, aligning the spatial resolution of the data with the network’s architectural requirements while maintaining critical anatomical information.

As a data augmentation technique for both datasets, we applied random flipping along the axial, coronal, and sagittal axes with a 0.5 probability, and by performing these flips at runtime during each training epoch—presenting each sample in multiple augmented forms—we enriched dataset variability, simulated diverse imaging conditions, and ultimately improved the model’s ability to generalize to unseen data.

### Segmentation and feature extraction: 3D SegResNet with latent space analysis

The segmentation task and feature extraction are performed using the 3D SegResNet architecture, available within the MONAI framework [28]. Pretrained on the BraTS2018 dataset, this network is specifically designed for brain tumor segmentation from MRI, producing voxel-wise segmentation maps of glioma subregions, such as necrosis, peritumoral edema, and contrast-enhancing tumor regions.

The network processes multimodal MRI inputs (T1, T1Gd, T2, T2-FLAIR) as separate channels, using complementary information to accurately segment the glioma subregion. The 3D SegResNet adopts a modified 3D U-Net architecture with residual connections, enhancing feature propagation and training stability. Its encoder-decoder structure captures hierarchical spatial features through downsampling and reconstructs segmentation masks via upsampling with skip connections. Residual blocks mitigate vanishing gradients, ensuring effective learning in deep networks.

#### 3D SegResNet fine-tuning

To address the domain change between the two public BraTS releases and assess performance in our private data set, we initialized the 3D SegResNet with weights pre-trained in BraTS2018 and subsequently fine-tuned the entire network on the BraTS2020 training set for 50 epochs (learning rate = 1 $\times$ e$^{-5}$, weight decay = 1 $\times$ 10$^{-5}$) using the same augmentation pipeline described above. Optimization was performed with Adam and a CosineAnnealingLR scheduler.

Inference was then carried out using these fine-tuned weights under three input configurations, in order to perform a fair comparison of our datasets:

- BraTS4CH: BraTS2020 data, 4-channel input (T1, T1Gd, T2, T2-FLAIR).
- BraTS2CH: BraTS2020 data, 2-channel input (T1 = 0, T1Gd, T2 = 0, T2-FLAIR).
- Besta2CH: IRCCS Besta data, 2-channel input (T1 = 0, T1Gd, T2 = 0, T2-FLAIR).

The dice similarity coefficient (DSC) was calculated for the three output channels: tumor core (TC), whole tumor (WT) and enhancement tumor (ET) across the validation splits. Table 4 reports the mean DSC values for each channel.

**Table 4:** Mean dice similarity coefficient for the output channels under the three input configurations

DSC channels	BraTS4CH	BraTS2CH	Besta2CH
DSC-TC	0.84	0.82	0.71
DSC-WT	0.90	0.88	0.78
DSC-ET	0.74	0.73	0.64
DSC-mean	**0**.**83**	**0**.**81**	**0**.**71**

*DSC* dice similarity coefficient, *TC* tumor core, *WT* whole tumor, *ET* enhancing tumor

Bold numbers indicate the best performance obtained among the three machine learning classifiers for each model

#### Principal component analysis

Beyond segmentation, this study uses the encoder’s latent space to extract high-dimensional features from the multimodal MRI inputs. These features provide a compact, abstract representation of the imaging data, capturing complex spatial and intensity patterns that are challenging to derive manually. After extracting the encoder latent features for each cohort, the principal component analysis (PCA) was applied independently to each dataset to retain the variance patterns specific to the cohort while reducing dimensionality. This step ensures efficient integration of encoder-derived features into the survival prediction pipeline, improving computational efficiency while preserving critical prognostic information.

By combining segmentation, feature extraction from the encoder’s latent space, and PCA-based dimensionality reduction, this approach provides a robust framework for both tumor characterization and survival prediction. The encoder of the 3D SegResNet generates 256 high-dimensional feature maps, each with spatial dimensions of 14 $\times$ 14 $\times$ 10. Each feature map represents a processed and abstract representation of the input data, emphasizing relevant patterns for the network’s task.

To reduce the dimensionality of these high-dimensional feature maps while retaining essential information, a summarization strategy was applied. Specifically, each volumetric feature map was condensed into a single scalar value by computing its median intensity along the spatial dimensions.

This approach preserves key information while minimizing redundancy and ensuring compact feature representation. The median was chosen because of its well-documented robustness to outliers, particularly in medical imaging data, where variability and noise are common challenges. It has been shown to effectively preserve diagnostic features, such as edges, while reducing noise and dimensionality, ensuring that essential information is retained for downstream analysis [29].

### OS classification

The dataset was randomly divided into a 70% training set—with an internal cross-validation loop for hyperparameter selection—and a 30% hold-out test set reserved for final evaluation of performance. Hyperparameter optimization was carried out using scikit-learn’s GridSearchCV [30], restricting all searches to the training folds in order to minimize overfitting, by applying a fivefold cross- validation. F1-score was adopted as scoring metric for hyperparameters selection.

Three classification algorithms were implemented for OS prediction:

1. *Random forest (RF):* RF works by constructing multiple decision trees during training and combining their outputs to enhance predictive accuracy and reduce overfitting. GridSearchCV was employed to explore variations in the number of trees, maximum depth, splitting criterion, minimum samples per split and leaf, features considered at each split, and class-weight balancing.
2. *XGBoost:* it is a gradient boosting algorithm known for its efficiency and accuracy in structured data classification. For XGBoost, a similar set of parameters was employed with the extension of the search to include learning rate and subsample fraction; during fitting, both training and validation losses were tracked and early stopping halted training after ten rounds without improvement.
3. *Multi-layer perceptron (MLP):* a deep learning model designed to capture complex interactions between input features. Hyperparameters were tuned over hidden-layer architectures, L2 regularization strengths, initial learning rates, and activation functions (relu, tanh, logistic). Early stopping—with 20% of the training data held out internally and a patience of ten epochs—prevented overtraining over a maximum of 1000 iterations.

Once optimal hyperparameters were identified for each algorithm, predictive performance on the independent test set was assessed by comparing paired F1 scores via the Wilcoxon signed-rank test, thereby providing a statistically robust evaluation of relative model efficacy.

## Experimental protocol

For the fine-tuning of our 3D SegResNet, we split the BraTS2020 dataset into a training set (70% of subjects) and a held-out validation set (30%), ensuring that each patient appeared in only one partition. The network has been initialized with weights pretrained on BraTS2018 and optimized all parameters using the Adam optimizer (initial learning rate = 1 $\times$ $10^{-5}$, weight decay = 1 $\times$ $10^{-5}$). To promote smooth convergence, we employed a CosineAnnealingLR scheduler over 50 epochs, gradually reducing the learning rate toward zero. Training proceeded for a fixed 50 epochs, and whenever the validation loss reached a new minimum, the model checkpoint was saved. This protocol provided a stable and reproducible adaptation of the pretrained backbone to the BraTS2020 domain.

We first applied principal component analysis (PCA) to the 256-dimensional latent feature vectors extracted from imaging data, retaining components that explained 90% of the cumulative variance. These principal components were then concatenated with patients’ age, which was standardized to zero mean and unit variance. To determine the optimal number of PCA components, we inspected scree plots for each cohort (BraTS4CH, BraTS2CH, and Besta2CH) and observed a clear knee point at approximately 40 components, beyond which additional components contributed predominantly noise. Accordingly, we defined a candidate interval centered around 40 components and performed an exhaustive grid search over this range. Survival prediction models—Random Forest, XGBoost, and a Multi-Layer Perceptron (MLP)—were then trained separately on each cohort, with exhaustive hyperparameter tuning via grid search confined to the training data.

The training and evaluation pipeline utilized a high-performance computing server equipped with a NVIDIA A100 GPU (40 GB memory), 4 CPU cores, and 64 GB of RAM. Model performance was assessed using metrics such as F1-score, AUC-ROC, accuracy, precision, recall and confusion matrices, providing a comprehensive evaluation across both datasets.

## Results

We present the comparative performance results for all three input configurations (BraTS4CH, BraTS2CH, Besta2CH) evaluated across three machine learning classifiers—Random Forest, XGBoost, and Multi-Layer Perceptron—using weighted and F1-score, AUC-ROC, and accuracy as primary metrics (Table 5).

To assess robustness and compare classifiers, we conducted paired Wilcoxon signed-rank tests on per-patient F1-scores between the MLP, RF, and XGBoost:

- BraTS4CH: MLP significantly outperformed RF (*p* = 0.005) and XGB (*p* = 0.005).
- BraTS2CH: MLP significantly outperformed RF (*p* = 0.01), while its difference with XGB was not significant.
- Besta2CH: MLP again beat RF significantly (*p* = 0.01) with no significant gap versus XGB.

**Table 5:** Performance metrics of machine learning models for overall survival prediction on BraTS2020 and IRCCS Besta datasets

Model	F1-score	AUC	Accuracy
*BraTS4CH model*			
Random forest classifier	0.65	0.68	0.64
XGBoost	0.70	0.71	0.67
MLP	**0**.**71**	**0**.**74**	**0**.**71**
*BraTS2CH model*			
Random forest classifier	0.63	0.68	0.61
XGBoost	0.62	0.64	0.62
MLP	**0**.**67**	**0**.**70**	**0**.**67**
*Besta2CH model*			
Random forest classifier	0.61	0.66	0.61
XGBoost	**0**.**65**	0.65	**0**.**66**
MLP	0.62	**0**.**70**	0.62

*AUC* area under the curve, *MLP* multi-layer perceptron

Bold numbers indicate the best performance obtained among the three machine learning classifiers for each model

## Conclusion

Reliable prediction of overall survival (OS) is critical for glioblastoma management, a highly aggressive and heterogeneous disease with limited therapeutic options. In this study, we integrated deep learning-derived imaging features from a pre-trained and fine-tuned 3D SegResNet with clinical data to predict OS on two independent cohorts (BraTS2020 and IRCCS Besta).

This study demonstrates the potential of integrating deep learning-derived imaging features with clinical data for OS prediction. By leveraging latent-space features extracted from a pre-trained 3D SegResNet and combining them with clinical variables such as patient age, the proposed framework achieves a balance of scalability and predictive accuracy. The use of median-based summarization and principal component analysis (PCA) ensured efficient dimensionality reduction while preserving key survival-related patterns, addressing the limitations of traditional radiomics approaches that depend heavily on manually engineered features. This automated pipeline offers a reproducible, scalable, and clinically adaptable solution for analyzing high-dimensional imaging data.

Across three input configurations—four-modality BraTS4CH, two-modality BraTS2CH, and two-modality Besta2CH—we evaluated Random Forest (RF), XGBoost (XGB), and a fully connected neural network (MLP), tuning hyperparameters via grid search on the weighted F1-score. In BraTS4CH, MLP achieved the best performance (F1 = 0.71, AUC= 0.74, Accuracy = 0.71) as well as for BraTS2CH (F1 = 0.67, AUC = 0.70, Accuracy = 0.67). In Besta2CH, XGB achieved the highest F1 score and precision (F1 = 0.65, AUC = 0.65, Accuracy = 0.66), while MLP achieved the best AUC (F1 = 0.62, AUC = 0.70, Accuracy = 0.62).

Paired Wilcoxon signed-rank tests on per-patient F1-scores confirmed the statistical robustness of these findings. MLP significantly outperformed RF in all three configurations and did not differ significantly from XGB in the two-channel settings (*p* > 0.05), indicating that while MLP is the clear winner under full-modality data, both MLP and XGB are equally viable when only T1Gd + T2-FLAIR inputs are available. These results demonstrated that the proposed framework has potentiality in managing heterogeneous MRI cohorts.

A notable limitation is that PCA was performed independently within each cohort, yielding cohort-specific feature spaces and preventing a definitive assessment of cross-dataset generalizability. Future work will focus on joint dimensionality-reduction or domain-adaptation techniques to harmonize feature representations, as well as in-depth features similarity measurements between BraTS2020 and Besta datasets, extending the model to multi-class survival stratification for finer prognostic grouping, and incorporate molecular/genetic biomarkers (e.g., MGMT methylation, IDH mutation status) to further improve predictive power and clinical relevance.

## References

[1] Ostrom QT, Cioffi G, Gittleman H, Patil N, Waite K, Kruchko C, Barnholtz-Sloan JS (2019) Primary brain and other central nervous system tumors diagnosed in the united states in 2012–2016. Neuro Oncol 21(5):1–100. 10.1093/neuonc/noz150 31675094 10.1093/neuonc/noz150 PMC6823730

[2] Liang S, Fan X, Zhao M, Shan X, Li W, Ding P, You G, Hong Z, Yang X, Luan G, Ma W, Yang H, You Y, Yang T, Li L, Liao W, Wang L, Wu X, Yu X, Zhang J, Mao Q, Wang Y, Li W, Wang X, Jiang C, Liu X, Qi S, Liu X, Qu Y, Xu J, Wang W, Song Z, Wu J, Liu Z, Chen L, Lin Y, Zhou J, Liu X, Zhang W, Li S, Jiang T (2019) Clinical practice guidelines for the diagnosis and treatment of adult diffuse glioma-related epilepsy. Cancer Med 8(10):4527–4535. 10.1002/cam4.2362 31240876 10.1002/cam4.2362 PMC6712518

[3] Hajianfar G, Haddadi Avval A, Hosseini SA, Nazarfi M, Oveisi M, Shiri I, Zaidi H (2023) Time-to-event overall survival prediction in glioblastoma multiforme patients using magnetic resonance imaging radiomics. Radiol Med 128(12):1521–1534. 10.1007/s11547-023-01725-3 37751102 10.1007/s11547-023-01725-3 PMC10700216

[4] Doniselli FM, Pascuzzo R, Agrò M, Aquino D, Anghileri E, Farinotti M, Pollo B, Paterra R, Cuccarini V, Moscatelli M, DiMeco F, Sconfienza LM (2023) Development of a radiomic model for MGMT promoter methylation detection in glioblastoma using conventional MRI. Int J Mol Sci 25(1):138. 10.3390/ijms25010138 38203308 10.3390/ijms25010138 PMC10778771

[5] Hegi ME, Diseren AC, Gorlia T, Hamou MF, Tribolet N, Weller M, Kros JM, Hainfellner JA, Mason W, Mariani L, Bromberg JE, Hau P, Mirimanoff RO, Cairncross JG, Janzer RC, Stupp R (2005) MGMT gene silencing and benefit from temozolomide in glioblastoma. N Engl J Med 352(10):997–1003. 10.1056/NEJMoa043331 15758010 10.1056/NEJMoa043331

[6] Turcan S, Rohle D, Goenka A, Walsh LA, Fang F, Yilmaz E, Campos C, Fabius AW, Lu C, Ward PS, Thompson CB, Kaufman A, Guryanova O, Levine R, Heguy A, Viale A, Morris LG, Huse JT, Mellinghoff IK, Chan TA (2012) IDH1 mutation is sufficient to establish the glioma hypermethylator phenotype. Nature 483(7390):479–483. 10.1038/nature10866 22343889 10.1038/nature10866 PMC3351699

[7] Molenaar RJ, Verbaan D, Lamba S, Zanon C, Jeuken JW, Boots-Sprenger SH, Wesseling P, Hulsebos TJ, Troost D, Tilborg AA, Leenstra S, Vandertop WP, Bardelli A, Noorden CJ, Bleeker FE (2014) The combination of IDH1 mutations and MGMT methylation status predicts survival in glioblastoma better than either IDH1 or MGMT alone. Neuro Oncol 16(9):1263–1273. 10.1093/neuonc/nou005 24510240 10.1093/neuonc/nou005 PMC4136888

[8] Combs SE, Rieken S, Wick W, Abdollahi A, Deimling A, Debus J, Hartmann C (2011) Prognostic significance of IDH-1 and MGMT in patients with glioblastoma: one step forward, and one step back? Radiat Oncol 6:115. 10.1186/1748-717X-6-115 21910919 10.1186/1748-717X-6-115 PMC3199258

[9] Shukla G, Alexander GS, Bakas SA, Nikam R, Talekar K, Palmer JD, Shi W (2017) Advanced magnetic resonance imaging in glioblastoma: a review. Chin Clin Oncol 6(4):4. 10.21037/cco.2017.06.28 28841802 10.21037/cco.2017.06.28

[10] Choi Y, Jang J, Kim BS, Ahn KJ (2023) Pretreatment MR-based radiomics in patients with glioblastoma: a systematic review and meta-analysis of prognostic endpoints. J Radiol 168:111130. 10.1016/j.ejrad.2023 10.1016/j.ejrad.2023.111130 37827087

[11] Bohman LE, Swanson KR, Moore JL, Mandigo C, Hankinson T, Assanah M, Canoll P, Bruce JN (2010) Magnetic resonance imaging characteristics of glioblastoma multiforme: implications for understanding glioma ontogeny. Neurosurgery 67(5):1319–1327. 10.1227/NEU.0b013e3181f556ab 20871424 10.1227/NEU.0b013e3181f556ab PMC3774031

[12] Itakura H, Achrol AS, Mitchell LA, Loya JJ, Liu T, Westbroek EM, Feroze AH, Rodriguez S, Echegaray S, Azad TD, Yeom KW, Napel S, Rubin DL, Chang SD, Harsh GR, Gevaert O (2015) Magnetic resonance image features identify glioblastoma phenotypic subtypes with distinct molecular pathway activities. Sci Transl Med 7(303):303–138. 10.1126/scitranslmed.aaa7582 10.1126/scitranslmed.aaa7582 PMC4666025 26333934

[13] Bae S, Choi YS, Ahn SS, Kang SG, Kim EH, Lee SK (2018) Radiomic MRI phenotyping of glioblastoma: improving survival prediction. Radiology 289(3):797–806. 10.1148/radiol.2018180200 30277442 10.1148/radiol.2018180200

[14] Li G, Li L, Li Y, Qian Z, Wu F, He Y, Jiang H, Li R, Wang D, Zhai Y, Wang Z, Jiang T, Zhang J, Zhang W (2022) An MRI radiomics approach to predict survival and tumour-infiltrating macrophages in gliomas. Brain 145(3):1151–1161. 10.1093/brain/awab340 35136934 10.1093/brain/awab340 PMC9050568

[15] Rizzo S, Botta F, Raimondi S, Origgi D, Fanciullo C, Morganti AG, Bellomi M (2018) Radiomics: the facts and the challenges of image analysis. Eur Radiol Exp 2(1):36. 10.1186/s41747-018-0068-z 30426318 10.1186/s41747-018-0068-z PMC6234198

[16] Woznicki P, Laqua FC, Al-Haj A, Bley T, Baeßler B (2023) Addressing challenges in radiomics research: systematic review and repository of open-access cancer imaging datasets. Insights Imaging 14:216. 10.1186/s13244-023-01556-w 38087062 10.1186/s13244-023-01556-w PMC10716101

[17] Visvikis D, Cheze Le Rest C, Jaouen V, Hatt M (2019) Artificial intelligence, machine (deep) learning and radio(geno)mics: definitions and nuclear medicine imaging applications. J Nucl Med Mol Imaging 46:2630–2637. 10.1007/s00259-019-04373-w 10.1007/s00259-019-04373-w 31280350

[18] He K, Zhang X, Ren S, Sun J (2016) Deep residual learning for image recognition. In: 2016 IEEE conference on computer vision and pattern recognition (CVPR), Las Vegas, NV, USA, pp 770–778. 10.1109/CVPR.2016.90

[19] Pei L, Vidyaratne L, Rahman MM, Iftekharuddin KM (2020) Context aware deep learning for brain tumor segmentation, subtype classification, and survival prediction using radiology images. Sci Rep 10(1):19726. 10.1038/s41598-020-74419-9 33184301 10.1038/s41598-020-74419-9 PMC7665039

[20] Chato L, Latifi S (2017) Machine learning and deep learning techniques to predict overall survival of brain tumor patients using MRI images. In: IEEE, pp 9–14

[21] Sun L, Zhang S, Chen H, Luo L (2019) Brain tumor segmentation and survival prediction using multimodal MRI scans with deep learning. Front Neurosci 13:810. 10.3389/fnins.2019.00810 31474816 10.3389/fnins.2019.00810 PMC6707136

[22] McKinley R, Rebsamen M, Daetwyler K, Meier R, Radojewski P, Wiest R (2020) Uncertainty-driven refinement of tumor-core segmentation using 3d-to-2d networks with label uncertainty. Springer, pp 565–573. arXiv:2012.06436

[23] Valbuena Rubio S, García-Ordás MT, García-Olalla Olivera O, Alaiz-Moretón H, González-Alonso MI, Benítez-Andrades JA (2023) Survival and grade of the glioma prediction using transfer learning. PeerJ Comput Sci 9:1723. 10.7717/peerj-cs.1723 10.7717/peerj-cs.1723 PMC10773899 38192446

[24] Myronenko A (2018) 3d MRI brain tumor segmentation using autoencoder regularization. arXiv:1810.11654

[25] Klein S, Staring M, Murphy K, Viergever MA, Pluim JPW (2010) elastix: a toolbox for intensity-based medical image registration. IEEE Trans Med Imaging 29:196–205. 10.1109/TMI.2009.2035616 19923044 10.1109/TMI.2009.2035616

[26] Isensee F, Schell M, Pflueger I, Brugnara G, Bonekamp D, Neuberger U, Wick A, Schlemmer HP, Heiland S, Wick W, Bendszus M, Maier-Hein KH, Kickingereder P (2019) Hd-bet: automated brain extraction of multisequence MRI using artificial neural networks. Neuroimage 40:4952–4964. 10.1002/hbm.24750 10.1002/hbm.24750 PMC6865732 31403237

[27] Lowekamp BC, Chen DT, Ibáñez L, Blezek D (2013) The design of simpleitk. Front Neuroinform 7:45. 10.3389/fninf.2013.00045 24416015 10.3389/fninf.2013.00045 PMC3874546

[28] MONAI consortium: 3D segmentation tutorial—MONAI GitHub repository (2023). https://github.com/Project-MONAI/tutorials/tree/main/3d_segmentation. Accessed: 27 Dec 2023

[29] Arias-Castro E, Donoho DL (2009) Does median filtering truly preserve edges better than linear filtering? 37:1172–1206. 10.1214/08-AOS604

[30] Pedregosa F, Varoquaux G, Gramfort A, Michel V, Thirion B, Grisel O, Blondel M, Prettenhofer P, Weiss R, Dubourg V, Vanderplas J, Passos A, Cournapeau D, Brucher M, Perrot M, Duchesnay E (2011) Scikit-learn: machine learning in python. J Mach Learn Res 12:2825–2830

